# Root-to-Shoot Hormonal Communication in Contrasting Rootstocks Suggests an Important Role for the Ethylene Precursor Aminocyclopropane-1-carboxylic Acid in Mediating Plant Growth under Low-Potassium Nutrition in Tomato

**DOI:** 10.3389/fpls.2016.01782

**Published:** 2016-11-29

**Authors:** Cristina Martínez-Andújar, Alfonso Albacete, Ascensión Martínez-Pérez, José Manuel Pérez-Pérez, María José Asins, Francisco Pérez-Alfocea

**Affiliations:** ^1^Centro de Edafologia y Biologia Aplicada del Segura (CSIC)Murcia, Spain; ^2^Instituto de Bioingeniería, Universidad Miguel Hernández, Edificio VinalopóAlicante, Spain; ^3^Instituto Valenciano de Investigaciones Agrarias (IVIA)Valencia, Spain

**Keywords:** grafting, micronutrients, phytohormones, recombinant inbred lines, root-to-shoot communication, *Solanum*

## Abstract

Selection and breeding of rootstocks that can tolerate low K supply may increase crop productivity in low fertility soils and reduce fertilizer application. However, the underlying physiological traits are still largely unknown. In this study, 16 contrasting recombinant inbred lines (RILs) derived from a cross between domestic and wild tomato species (*Solanum lycopersicum* × *Solanum pimpinellifolium*) have been used to analyse traits related to the rootstock-mediated induction of low (*L, low shoot fresh weight*) or high (*H, high shoot fresh weight*) vigor to a commercial F1 hybrid grown under control (6 mM, *c*) and low-K (1 mM, *k*). Based on hormonal and ionomic composition in the root xylem sap and the leaf nutritional status after long-term (7 weeks) exposure low-K supply, a model can be proposed to explain the rootstocks effects on shoot performance with the ethylene precursor aminocyclopropane-1-carboxylic acid (ACC) playing a pivotal negative role. The concentration of this hormone was higher in the low-vigor *Lc* and *Lk* rootstocks under both conditions, increased in the sensitive *HcLk* plants under low-K while it was reduced in the high-vigor *Hk* ones. Low ACC levels would promote the transport of K *vs*. Na in the vigorous *Hk* grafted plants. Along with K, Ca, and S, micronutrient uptake and transport were also activated in the tolerant *Hk* combinations under low-K. Additionally, an interconversion of *trans*-zeatin into *trans*-zeatin riboside would contribute to decrease ACC in the tolerant *LcHk* plants. The high vigor induced by the *Hk* plants can also be explained by an interaction of ACC with other hormones (cytokinins and salicylic, abscisic and jasmonic acids). Therefore, *Hk* rootstocks convert an elite tomato F1 cultivar into a (micro) nutrient-efficient phenotype, improving growth under reduced K fertilization.

## Introduction

Potassium (K) is the most abundant cation in plant tissues and is required for plant development and crop yield (Wang and Wu, 2013). K deficiency is associated with lowered photosynthesis, transpiration rates, and stomatal conductance (Kanai et al., 2011), whereas photorespiration and respiration are stimulated (Singh and Blanke, 2000). However, most plants can survive under low-K conditions, largely because of the high-affinity K uptake system in the roots which is induced under K deficiency (Armengaud et al., 2004; Chérel et al., 2014; Hafsi et al., 2014).

K starvation not only triggers an upregulation of K transporters, but also involves changes in different signaling molecules including reactive oxygen species (ROS), Ca and several phytohormones such us ethylene, jasmonic acid (JA), and auxins (Armengaud et al., 2004; Shin and Schachtman, 2004; Cao et al., 2006; Hafsi et al., 2014). Ethylene signaling is a key component of the plant response to low K that stimulates the production of ROS and induces changes in root morphology, and gene expression of high affinity transporters (Jung et al., 2009). In turn, many studies suggest that ethylene acts in conjunction with other hormones and signaling molecules to regulate those responses (García et al., 2015). Although recent transcriptomic studies have revealed that many JA-related genes, including JA biosynthesis genes, are induced in response to low K stress (Ruan et al., 2015), the relationship between K deficiency and the JA pathway is still poorly characterized (Armengaud et al., 2004; Fan et al., 2014; Ruan et al., 2015). Furthermore, little is known about the roles of abscisic acid (ABA), salicylic acid (SA), gibberellins (GAs), and cytokinins (CKs) under K deficiency.

The K requirement for optimal plant growth is in the range of 2–5% of the plant dry weight of the vegetative parts and fleshy fruits (Marschner, 1995). In greenhouse tomatoes, 2 mM is the minimum K concentration required to prevent growth reduction and visible deficiency symptoms, while at least 5 mM is necessary to produce maximum fruit yield (Besford and Maw, 1975; Atherton and Rudich, 1986; Kanai et al., 2007, 2011). Although gaining insights into the regulatory mechanisms of plant responses to K deficiency in Arabidopsis may lead to improvements in K utilization efficiency (KUE), the transfer of this knowledge into crop species through conventional breeding or biotechnological approaches is still a scientific challenge that need to be addressed in order to implement a “more-with-less” agriculture. As alternative, grafting provides a tool to explore and exploit new and existing genetic variability through regulating root-shoot interactions governing a range of physiological responses, including tolerance to abiotic stresses such as drought, salinity, and nutrient deficiency (Ghanem et al., 2011; Pérez-Alfocea, 2015; Albacete et al., 2015a). Grafting can enhance nutrient uptake and/or utilization efficiency in vegetables (Rouphael et al., 2008b; Colla et al., 2010; Salehi et al., 2010; Savvas et al., 2010), thus contributing to reduce the amount of fertilizers and production costs. Enhancement of K uptake through grafting has been reported in eggplant (Leonardi and Giuffrida, 2006), melon (Qi et al., 2006), watermelon (Huang et al., 2013), mini watermelon (Rouphael et al., 2008a; Huang et al., 2013), cucumber (Zhu et al., 2008), pepper (Penella et al., 2015), and tomato (Albacete et al., 2009; Huang et al., 2013; Schwarz et al., 2013). However, the mechanisms underlying the rootstock-mediated growth improvement in response to K deprivation have not been elucidated. The availability of RIL populations derived from crosses between the cultivated tomato *Solanum lycopersicum* and wild species such as *Solanum pimpinellifolium*, provides a powerful tool to identify the physiological and genetic determinants of the rootstock-mediated improvement of crop performance (Albacete et al., 2009, 2015a; Estañ et al., 2009; Asins et al., 2010). In the present study, we selected four groups of contrasting RILs on the basis of their high (*H*) or low (*L*) induced vigor to the scion under control (*c*) and low K (*k*) conditions to gain insights into the hormonal and nutritional components involved in the rootstock-mediated adaptation to low-K supply. This knowledge could be useful for further KUE improvement in tomato and other species.

## Materials and methods

### Plant material and growth conditions

This study formed part of a larger experiment in which 114 RILs derived from a cross between *S. lycopersicum* × *S. pimpinellifolium* (Monforte et al., 1997) segregating for salinity resistance (Estañ et al., 2009) were used as rootstocks of the commercial tomato hybrid cv. Boludo F1 (Seminis Vegetable Seeds Ibérica S.A., Barcelona, Spain) used as the scion to search for tolerant rootstocks under K deficiency (Albacete et al., 2015b). Grafting was performed using the splicing method at the two to three true leaf stages where the scion was attached at the first node of the rootstock (Savvas et al., 2011). One month later (25th September 2012), grafted plants were transferred to a commercial greenhouse located in the Mazarrón tomato producing area (37°33′19.96″ N, 1° 22′53.95″ W) and cultivated in a semi-hydroponic system using sand as substrate. In this study, 16 grafted RILs were phenotypically selected for the vigor induced to the scion (measured as shoot fresh weight, SFW) and classified into four groups (12 plants per group) on the basis of their growth response to each treatment: the first group comprised four rootstocks (RILs 27, 60, 267, and 240) having low vigor (low SFW) irrespective of K treatment (*LcLk*); the second group was four rootstocks (1, 62, 204, and 148) showing high vigor under *c* and low vigor under *k* conditions (*HcLk*); the third group consisted of four rootstocks (47, 132, 167, and 209) with low vigor under *c* and high vigor under *k* conditions (*LcHk*); and finally a fourth group comprising four rootstocks with high vigor regardless of treatment (*HcHk*; Figure S1).

Three plants per graft combination were randomly distributed and irrigated with a standard (Cadahia et al., 1995; 6 mM K, control) or modified (1 mM K, low-K) nutrient solution for a period of 7 weeks. The concentration of the other macro and micronutrients in both standard and modified nutrient solutions were: N, 12.5 mM (NO_3_:NH_4_, 12:0.5); P, 1.5 mM; Ca, 4 mM; Mg, 2 mM; Fe, 100 μM; B, 46 μM; Mn, 9 μM; Zn, 0.76 μM; Cu, 0.75 μM and Mo 0.02 μM.

Forty-eight days after starting the low-K treatment, the second fully expanded mature leaf of 3 plants per graft combination was weighted and used for leaf area and physiological determinations. Leaf area was determined using an LI-3100AC Area Meter (LI-Cor, Lincoln, NE, USA). Shoot and roots were detached and immediately weighed. The plants were severed 1.5 cm above the graft union. A short length silicone tube was fitted to collect the spontaneous xylem sap exudate which was removed by means of pipette, placed in pre-weighed Eppendorf tube, and immediately frozen on liquid nitrogen and stored at −80°C until analysis. Sap volume and exudation time were recorded in order to calculate the sap flow rate (Netting et al., 2012) and nutrient and hormone delivery to the shoot (sap flow × analyte concentration in the xylem).

### Chlorophyll fluorescence

Modulated chlorophyll fluorescence was measured in dark adapted (30 min) leaves in the second fully expanded leaf leaflet in 3 plants per graft combination (12 in total per contrasting group), using a chlorophyll fluorimeter OS-30 (OptiSciences, Herts, UK) with an excitation source intensity of 3000 mmol m^−2^ s^−1^. The minimal fluorescence intensity (*F0*) in a dark-adapted state was measured in the presence of a background far-red light. The maximal fluorescence intensity in the dark-adapted state (*Fm*) was measured by 0.8 s saturating pulses (3000 mmol m^−2^ s^−1^). The maximum quantum yield of open photosystem II (PSII) (*Fv/Fm*) was calculated as *(Fm*−*F*_0_*)/Fm* (Maxwell and Johnson, 2000).

### Ion concentration

For ionic quantification, fresh tissue samples were oven dried for 48 h at 80°C, weighed (dry weight) and digested in a HNO_3_:HClO (2:1, v/v) solution. Ion analysis was conducted in root xylem sap and leaf tissue samples with inductively coupled plasma spectrometry (ICP-OES, Thermo ICAP 6000 Series).

### Hormone analysis

Cytokinins (Z; ZR; and iP), ACC, ABA, JA, SA, and gibberellins (GA_1_, GA_3_, and GA_4_) were analyzed according to Albacete et al. (2008) with some modifications. Briefly, xylem sap samples were filtered through 13 mm diameter Millex filters with 0.22 μm pore size nylon membrane (Millipore, Bedford, MA, USA). Ten microliters of filtrated extract were injected in a U-HPLC-MS system consisting of an Accela Series U-HPLC (ThermoFisher Scientific, Waltham, MA, USA) coupled to an Exactive mass spectrometer (ThermoFisher Scientific, Waltham, MA, USA) using a heated electrospray ionization (HESI) interface. Mass spectra were obtained using the Xcalibur software version 2.2 (ThermoFisher Scientific, Waltham, MA, USA). For quantification of the plant hormones, calibration curves were constructed for each analyzed component (1, 10, 50, and 100 μg l^−1^).

### Statistics

Analysis of variance, correlation analyses, and principal component analysis (PCA) were performed using SPSS for Windows (Version 22.0, SPSS Inc., Chicago, IL, USA). Means of different graft combinations were compared using Tukey's test at 0.05 of confidence level and the Varimax method was used for PCA.

## Results

### Rootstock-mediated variation in shoot vigor and chlorophyll fluorescence

As reported previously in Albacete et al. (2015b), the whole RIL population used as rootstocks induced 2.5-fold variability in SFW in the commercial tomato hybrid cv. Boludo F1 grown under low-K supply. About 15% of rootstock genotypes significantly increased or decreased SFW of the commercial scion with respect to the self-grafted Boludo F1 plants. As shown in Figure S1, a weak but significant linear correlation exists in shoot biomass production between control and low-K conditions when the RIL population was used as rootstock. To carry out the present study, four contrasting groups (4 lines per group) of rootstocks were selected for their differential effect on shoot biomass under control and low-K supply (See Material and Methods): *LcLk, HcLk, LcHk*, and *HcHk*.

**Figure 1:**
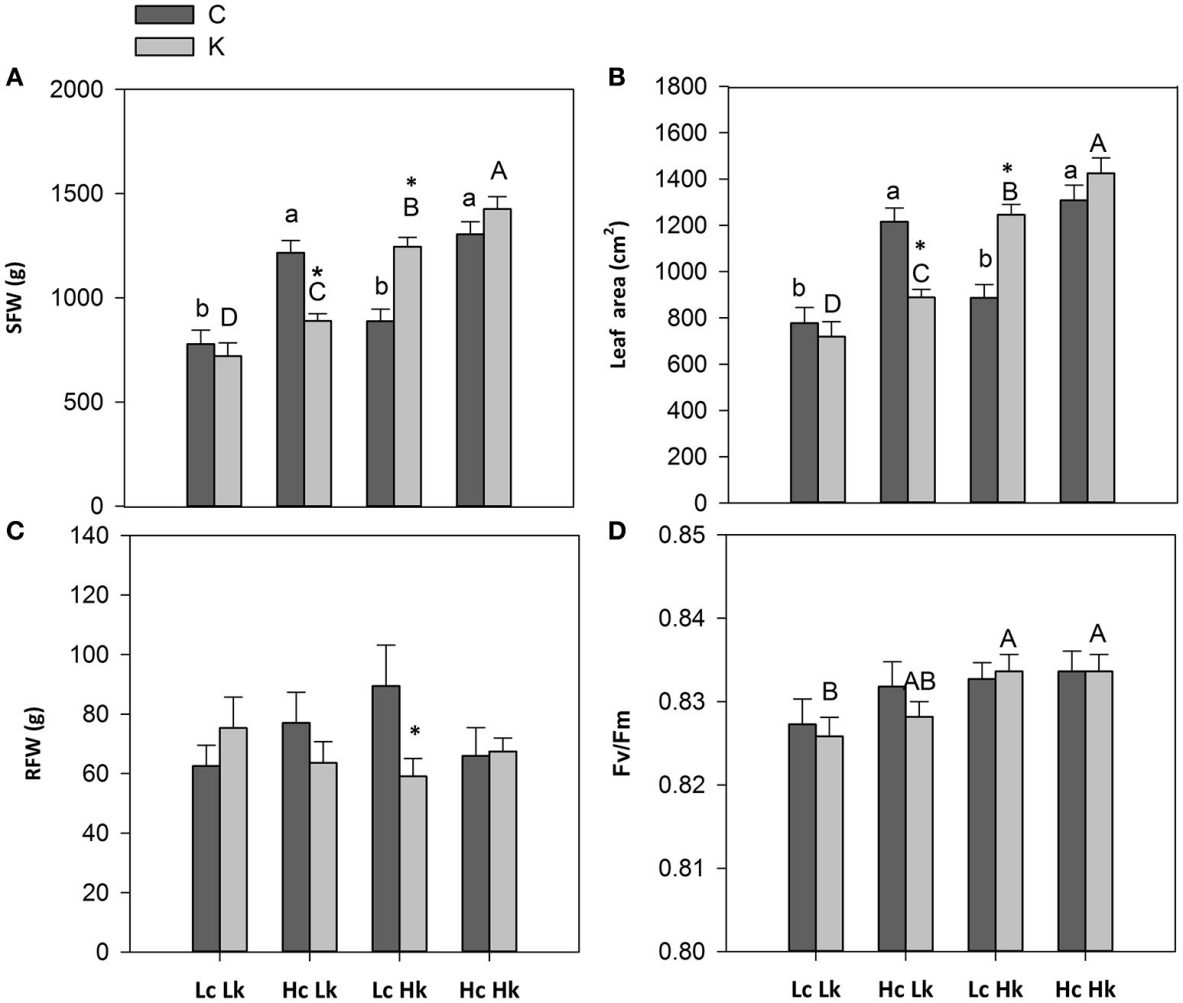
**Shoot fresh weight (SFW) (A)**, leaf area (LA) **(B)**, root fresh weight (RFW) **(C)**, and chlorophyll fluorescence (F_v_/F_m_) **(D)** of the scion (*Solanum lycopersicum* cv. Boludo F1) grafted onto a population of recombinant inbred lines (RILs) from a cross between *Solanum lycopersicum* × *Solanum pimpinellifolium* with high (*H*) or low (*L*) vigor growing under control (*c*) and low (*k*) conditions during 48 days. Different letters indicate significant differences among graft combinations (*n* = 12, *P* < 0.05) within each treatment. ^*^indicate significant differences between control and low-K treated plants according to the Tuckey test (*P* < 0.05).

Shoot biomass of the non-grafted scion was reduced by 30% under low-K compared to control conditions (Albacete et al., 2015a). The *H* rootstocks produced 40–50% more SFW than the *L* ones, while low-K decreased (*HcLk*) or increased (*LcHk*) shoot biomass by 25–30%, compared to control conditions (Figure 1A, Table S1). A similar effect of the rootstock was observed for leaf area (Figure 1B) and leaf biomass (data not shown). In contrast, root biomass was not affected by the rootstock genotype, while only the *LcHk* plants registered a significant decrease in root biomass under low-K compared with control conditions (Figure 1C). The *F*_*v*_*/F*_*m*_ after 48 days of treatment was significantly higher in the *Hk* than in the *Lk* plants under low-K, but this parameter was not affected by the low-K supply compared to the control (Figure 1D).

### Principal component analysis of rootstock-mediated response under low-K

**Figure 2:**
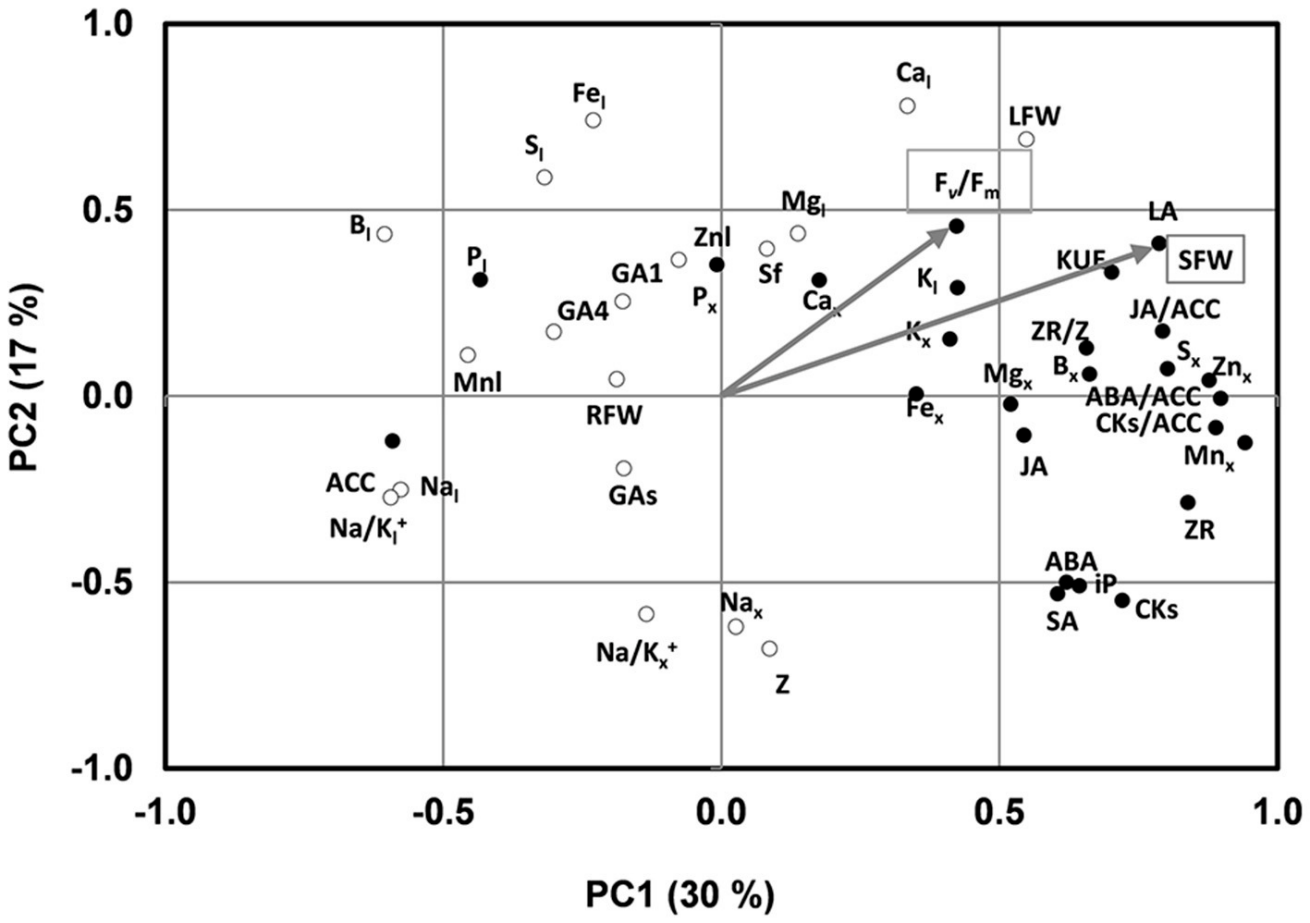
**Two axes of a principal component (PC1, PC2) analysis under low K conditions showing shoot fresh weight (SFW) and ***F***_**v**_*/**F***_**m**_ trait vectors (indicated by arrow) and the position of all variables (denoted by abbreviations) studied under low K conditions**. Hormonal parameters in the xylem sap: ZR, Z, iP, CKs, ACC, ABA, JA, SA, GA_1_, GA_4_, GAs; Hormonal ratios: ZR/Z, CKs/ACC, ABA/ACC, JA/ACC; Ionic parameters in the xylem sap: K_x_, Na_x_,P_x_, Mg_x_, S_x_, Ca_x_, Na_x_, Zn_x_, Mn_x_, Fe_x_, B_x_, Na/K_x_; ionic parameters in the leaf: K_l_, Na_l_, P_l_, Mg_l_, S_l_, Zn_l_, Mn_l_, Fe_l_, B_l_, Na/K_l_; biomass related parameters and others: SFW, shoot fresh weight; RFW, root fresh weight; LFW, leaf fresh weight; LA, leaf area; sf, sap flow; KUE, K use efficiency. The black and white circles indicate the variables belong to the cluster 1 and 2, respectively.

Under low-K supply, the growth parameters and *F*_v_*/F*_m_ covaried with most of the nutrients analyzed in the xylem sap along PC1, which explained 30% of the variability, especially the micronutrients Zn, Mn, and B, as well as the K concentration in the leaves (Figure 2). In contrast the growth parameters were negatively associated with the leaf concentration of Mn, B, P, S, and Na. Moreover, the root biomass covaried with the shoot growth-related parameters under control conditions (data not shown) but not under low-K conditions, suggesting that root growth was not a determinant of scion vigor under the low nutrient supply in the tested conditions.

Regarding the hormone-related parameters, ZR, ZR/Z, CK/ACC, GA_1_, and GA_4_ covaried with shoot and leaf growth under control conditions, while total CKs, iP, ABA, SA, JA, total Gas, and the ratio ABA/ACC were associated in an opposed cluster [data not shown). However, under low-K conditions, most hormonal parameters (total CKs, ZR, iP, ABA, SA, JA, CK/ACC, ABA/ACC and particularly JA/ACC, and ZR/Z)] clustered with shoot vigor, while the ethylene precursor ACC clustered in opposite direction (Figure 2).

### Root xylem sap flow

No differences were observed in the xylem sap flow among graft combinations in either control or low-K conditions. Under low-K only the *LcLk* plants registered a significant 2-fold increase in sap flow compared with control K conditions (Figure S2).

### Ionome in root xylem sap and leaf

#### Potassium and potassium use efficiency (KUE)

**Figure 3:**
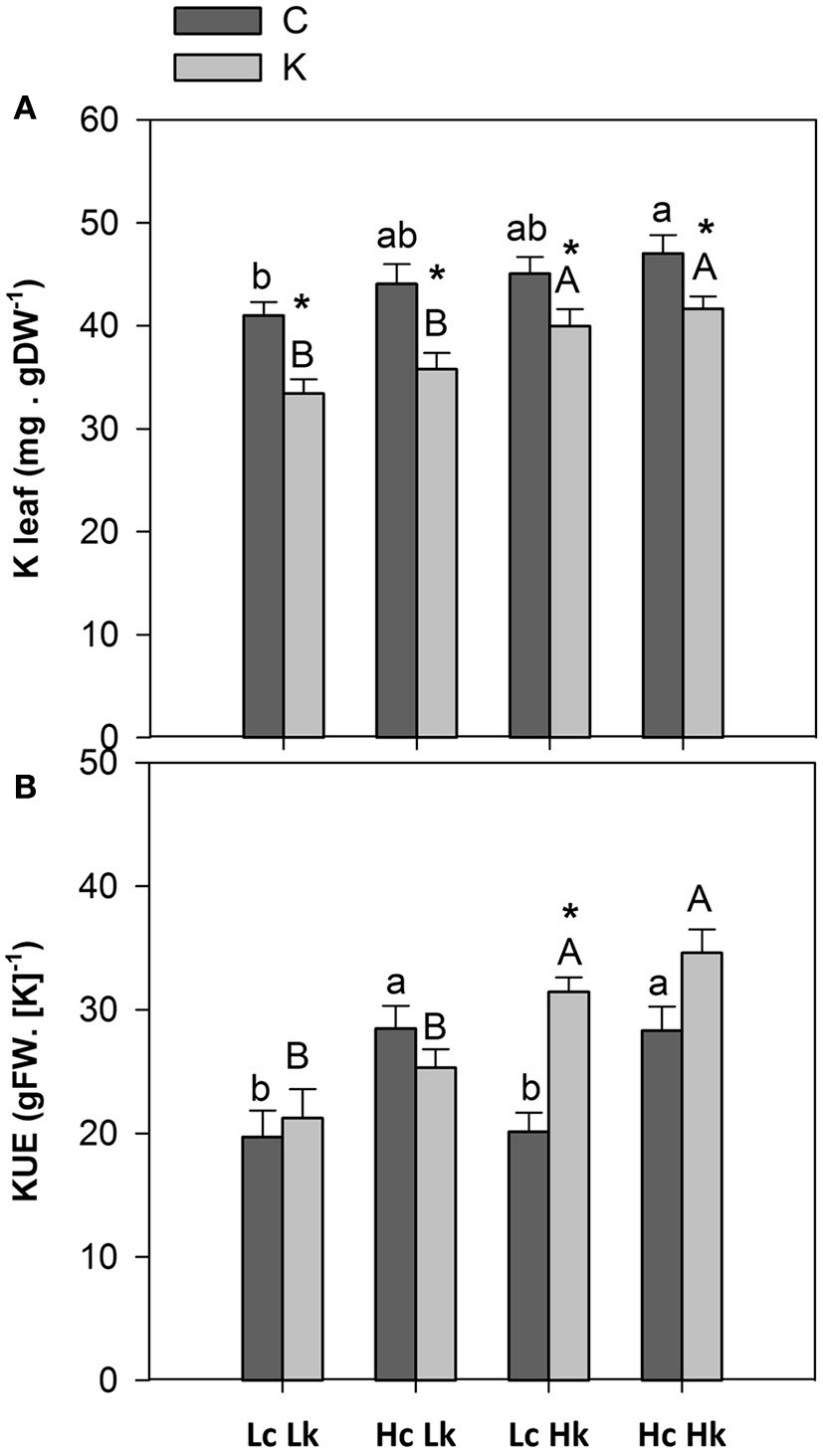
**Leaf potassium concentration (A)** and potassium use efficiency (KUE, calculated as the shoot biomass generated per unit of K assimilated by a photosynthetic mature leaf) **(B)** of the scion (*Solanum lycopersicum* cv. Boludo F1) grafted onto a population of recombinant inbred lines (RILs) from a cross between *Solanum lycopersicum* × *Solanum pimpinellifolium* with high (*H*) or low (*L*) vigor growing under control (*c*) and low K (*k*) conditions during 48 days. Different letters indicate significant differences among graft combinations (*n* = 12, *P* < 0.05) within each treatment. ^*^ indicate significant differences between control and low-K treated plants according to the Tuckey test (*P* < 0.05).

K concentration in the root xylem sap was not significantly affected by the graft combination or the K treatment (Table 1). However, leaf K concentration was significantly affected by both factors (Table S1), with the vigorous *HcHk* rootstocks registering 3% higher K concentrations than the low vigor *LcLk* ones under control K nutrition (Table 1, Figure 3A). Under low-K despite no reduction in the xylem sap K levels, leaf K concentration significantly decreased 10–20% compared with control plants. Nevertheless, the *Hk* rootstocks maintained a 15% higher leaf K concentration under low-K than the *Lk* ones (Figure 3A). This observation suggests that high rootstock-mediated vigor under low K supply (but not under control conditions) is related to a better ability of maintain high K concentration in the leaves. *This is supported by the positive correlation between SFW and leaf K concentration under low-K conditions (Table 2)*.

**Table 1:** **Potassium (K), sodium (Na), Na/K ratio, phosphorous, (P), iron (Fe), magnesium (Mg), sulphur (S), calcium (Ca), zinc (Zn), manganese (Mn), boron (B) in root xylem sap and leaf of the scion (***Solanum lycopersicum*** cv. Boludo F1) grafted onto a population of recombinant inbred lines (RILs) from a cross between ***Solanum lycopersicum*** × ***Solanum pimpinellifolium*** with high (H) or low (L) vigour growing under control (c) and low K (k) conditions during 48 days**.

			**Control**		**Low-K**	
			**Xylem sap (mg.l^−1^)**	**Leaf (mg.gDW^−1^)**	**Xylem sap (mg.l^−1^)**	**Leaf (mg.gDW^−1^)**
Macronutrients	K	LcLk	426.97 ± 51.05	41.01 ± 1.32b	403.20 ± 27.24	33.43 ± 1.39b^*^
		HcLk	386.19 ± 40.56	44.08 ± 1.90ab	396.38 ± 18.15	35.78 ± 1.57b^*^
		LcHk	483.65 ± 41.12	45.08 ± 1.62ab	431.84 ± 63.89	39.98 ± 1.66a^*^
		HcHk	441.41 ± 31.75	47.04 ± 1.78a	384.71 ± 35.63	41.67 ± 1.19a^*^
	Na	LcLk	40.88 ± 4.43	4.63 ± 0.51	46.38 ± 7.15	5.73 ± 0.67a^*^
		HcLk	32.81 ± 5.53	4.07 ± 0.37	42.32 ± 5.38	4.12 ± 0.21b
		LcHk	36.62 ± 3.91	3.48 ± 0.29	42.35 ± 9.55	3.27 ± 0.15b
		HcHk	35.06 ± 4.42	3.65 ± 0.41	40.88 ± 5.69	3.70 ± 0.22b
	Na/K	LcLk	0.11 ± 0.02	0.12 ± 0.09	0.12 ± 0.02	0.17 ± 0.03a^*^
		HcLk	0.11 ± 0.03	0.09 ± 0.01	0.11 ± 0.01	0.12 ± 0.01b
		LcHk	0.08 ± 0.01	0.08 ± 0.01	0.08 ± 0.02	0.08 ± 0.02b
		HcHk	0.08 ± 0.01	0.08 ± 0.01	0.14 ± 0.05	0.09 ± 0.01b
	P	LcLk	80.70 ± 20.98	5.90 ± 0.64	75.42 ± 11.63	8.48 ± 0.25^*^
		HcLk	81.93 ± 9.48	6.88 ± 0.25	86.61 ± 12.33	7.59 ± 0.58
		LcHk	66.97 ± 11.37	7.04 ± 0.31	91.39 ± 13.60	7.47 ± 0.30
		HcHk	70.95 ± 7.77	7.12 ± 0.45	75.99 ± 10.75	7.49 ± 0.21
	Mg	LcLk	45.62 ± 10.32	8.76 ± 0.70	38.16 ± 4.10	8.86 ± 0.56
		HcLk	37.63 ± 4.68	7.93 ± 0.61	39.08 ± 4.64	8.27 ± 0.39
		LcHk	38.19 ± 7.34	7.95 ± 025	41.13 ± 5.60	8.99 ± 0.42
		HcHk	45.63 ± 13.76	9.13 ± 0.55	39.69 ± 3.35	8.67 ± 0.33
	S	LcLk	71.63 ± 16.69	25.28 ± 3.59	54.11 ± 5.77	29.26 ± 3.00
		HcLk	72.58 ± 11.60	25.87 ± 2.58	68.62 ± 13.13	27.06 ± 2.92
		LcHk	58.78 ± 8.75	21.43 ± 2.01	70.07 ± 9.09	33.66 ± 2.89^*^
		HcHk	95.39 ± 28.78	26.54 ± 2.50	73.51 ± 9.95	30.91 ± 4.64
	Ca	LcLk	190.10 ± 49.21	34.67 ± 4.27b	135.80 ± 23.92	38.17 ± 3.11b
		HcLk	203.95 ± 33.05	34.97 ± 3.09b	173.55 ± 30.75	39.19 ± 2.84b^*^
		LcHk	147.83 ± 26.83	35.16 ± 1.48b	177.85 ± 25.73	43.14 ± 1.47ab^*^
		HcHk	166.06 ± 24.07	46.22 ± 2.48a	150.44 ± 23.13	47.96 ± 2.95a
Micronutrients	Zn	LcLk	0.33 ± 0.06	0.15 ± 0.02	0.35 ± 0.06b	0.17 ± 0.14
		HcLk	0.29 ± 0.05	0.17 ± 0.02	0.25 ± 0.04b	0.16 ± 0.01
		LcHk	0.51 ± 0.12	0.18 ± 0.01	0.43 ± 0.05b	0.16 ± 0.01
		HcHk	0.37 ± 0.05	0.16 ± 0.01	1.15 ± 0.32a^*^	0.18 ± 0.06
	Mn		1.35 ± 0.42	0.68 ± 0.06	1.38 ± 0.32ab	0.56 ± 0.09
		HcLk	0.72 ± 0.14	0.54 ± 0.07	1.16 ± 0.20b	0.49 ± 0.09
		LcHk	1.31 ± 0.40	0.46 ± 0.08	1.74 ± 0.38ab	0.63 ± 0.07
		HcHk	1.30 ± 0.21	0.62 ± 0.06	2.54 ± 0.56a^*^	0.48 ± 0.08
	Fe	LcLk	0.70 ± 0.16	0.29 ± 0.06	0.32 ± 0.04b	0.25 ± 0.02
		HcLk	0.28 ± 0.04	0.23 ± 0.03	0.38 ± 0.07ab	0.29 ± 0.04
		LcHk	0.76 ± 0.27	0.27 ± 0.03	0.60 ± 0.16a	0.34 ± 0.04
		HcHk	0.44 ± 0.06	0.33 ± 0.04	0.36 ± 0.04ab	0.29 ± 0.04
	B	LcLk	0.45 ± 0.24	0.07 ± 0.07	0.37 ± 0.09b	0.11 ± 0.02^*^
		HcLk	0.35 ± 0.10	0.09 ± 0.01	0.36 ± 0.10b	0.09 ± 0.08
		LcHk	0.33 ± 0.11	0.08 ± 0.01	0.54 ± 0.14ab	0.11 ± 0.00^*^
		HcHk	0.32 ± 0.09	0.09 ± 0.03	0.80 ± 0.17a^*^	0.10 ± 0.00

*Different letters indicate significant differences among graft combinations (n = 12, P < 0.05) within each treatment*.

* *indicate significant differences between control and low-K treated plants according to the Tuckey test (P < 0.05)*.

**Table 2:** **Linear correlation coefficients between shoot fresh weight (SFW) and ionomic and hormonal related parameters in the root xylem sap of the scion (***Solanum lycopersicum*** cv. Boludo F1) grafted onto a population of recombinant inbred lines (RILs) from a cross between ***Solanum lycopersicum*** × ***Solanum pimpinellifolium*** with high (H) or low (L) vigour growing under control (c) and low K (k) conditions during 48 days**.

**Ionic Parameters**												
**Macronutrients**								**Macronutrients**				
	**K**	**Na**	**Na/K**	**P**	**Mg**	**S**	**Ca**	**Zn**	**Mn**	**Fe**	**B**	
**XYLEM SAP**												
C	−0.075	−0.121	−0.029	0.205	0.109	0.274	0.199	−0.058	0.036	−0.256	−0.176	
K	0.097	−0.089	−0.016	0.023	0.123	0.265	0.077	0.487^**^	0.414^**^	0.105	0.585^**^	
**LEAF**												
C	0.045	0.001	0.005	0.260	−0.165	0.360^*^	0.208	0.090	0.149	−0.136	0.364^*^	
K	0.514^**^	−0.458^**^	−0.487^**^	−0.125	0.043	0.011	0.364^*^	0.117	−0.052	0.172	0.364^*^	
**Hormonal Parameters**												
	**ZR**	**Z**	**iP**	**CKs**	**ACC**	**ABA**	**SA**	**JA**	**ZR/Z**	**CKs/ACC**	**ABA/ACC**	**JA/ACC**
**XYLEM SAP**												
C	0.250	−0.494^**^	0.080	−0.349*^*^	−0.304	−0.471^*^	0.196	−0.224	0.466^**^	0.139	0.027	−0.05
K	0.179	−0.145	0.386^**^	0.262	−0.557^**^	0.263	0.402^**^	0.153	0.318^*^	0.487^**^	0.679^**^	0.627^**^
	**ZR**_f_	**iP**_f_	**CKs**_f_	**SA**_f_								
C	0.171	0.108	0.027	0.099								
K	0.358^*^	0.334^*^	0.319^*^	0.325^*^								

* *P < 0.05*,

** *P < 0.01, n = 48. K, potassium; Na, sodium; Na/K ratio; P, phosphorous; Mg, magnesium; S, sulphur; Ca, calcium; Zn, zinc; Mn, manganese; Fe, iron; B, boron. ZR, zeatin riboside; Z, zeatin; iP, isopentenyladenine; CKs, total cytokinins; ACC, 1-Aminocyclopropane-1-carboxylic acid; ABA, abscisic acid; SA, salicylic acid; JA, jasmonic acid; ZR/Z ratio; CKs/ACC ratio; JA/ACC ratio; ZR_f_, zeatin riboside flow rate; iP_f_, Isopentenyladenine flow rate; CKs_f_, total cytokinins flow rate; SA_f_, salicylic acid flow rate. Bold means the correlation values are significatives*.

KUE, calculated as the shoot biomass generated per unit of K assimilated by a photosynthetic mature leaf, was 30–40% higher in the most vigorous *HcHk* rootstocks than in the less vigorous *LcLk* ones under both growing conditions (Figure 3B). Interestingly, a 35% increase in KUE was registered in the tolerant *LcHk* graft combinations under low-K.

#### Sodium and Na/K ratio

Curiously, Na concentration of in root xylem sap and leaves was higher in the low-vigor *LcLk* rootstocks than in other graft combinations (Table 1) Moreover, Na concentrations tended to increase in the xylem sap in all graft combinations under low-K. Na concentration and Na/K ratio significantly increased in the leaves of the low-vigor *LcLk* plants under low-K (Table 1).

#### Phosphorous, magnesium, sulfur, and calcium

Non-significant differences in P, Mg, and S concentrations were found in both in root xylem sap and leaves among graft combinations under both low and control K supply (Table 1). Curiously, the *LcHk* rootstocks registered the highest increase (but not significant) in P (only in xylem sap), Mg (in both xylem sap and leaf), and S (only in leaf) concentrations at low-K supply with respect to normal conditions (Table 1).

Non-significant differences were found in Ca concentration in the root xylem sap among plants grafted onto contrasting rootstocks (Table 1). However, leaf Ca concentration was 20–25% higher in the vigorous *HcHk* plants than in the low vigor (*Lc* and *Lk*) ones under both growing conditions. Interestingly, leaf Ca concentration was well correlated with SFW under low-K supply (Table 2).

Plants grafted onto the tolerant *LcHk* rootstocks registered the lowest P, S, and Ca concentrations in the root xylem sap under control K nutrition and the highest K, P, Mg, S, and Ca concentrations under low-K supply (Table 1), suggesting that the low vigor of those plants under normal K fertilization was due to rootstock-mediated interference with the loading of nutrients into the xylem (sap flow was similar), which was bypassed under low-K nutrition.

#### Micronutrients

The vigorous *HcHk* grafted plants registered 2.7–5 (Zn) and 1.5–2.2 (Mn, B) -fold higher micronutrient concentration in the root xylem sap under low-K, compared to the other rootstocks (Table 1). The tolerant *LcHk* plants registered intermediate values between the *LcLk* and the *HcHk* plants for those nutrients and 1.6 to 1.9-fold higher Fe concentration in the xylem sap under low-K supply. However, non-significant differences between different graft combinations were found in the leaves. Importantly, a positive correlation was found between Zn, Mn, B concentration in the rootstock xylem sap and SFW of the scion under low-K conditions (Table 2).

### Hormone concentrations in root xylem sap

#### Cytokinins

**Figure 4:**
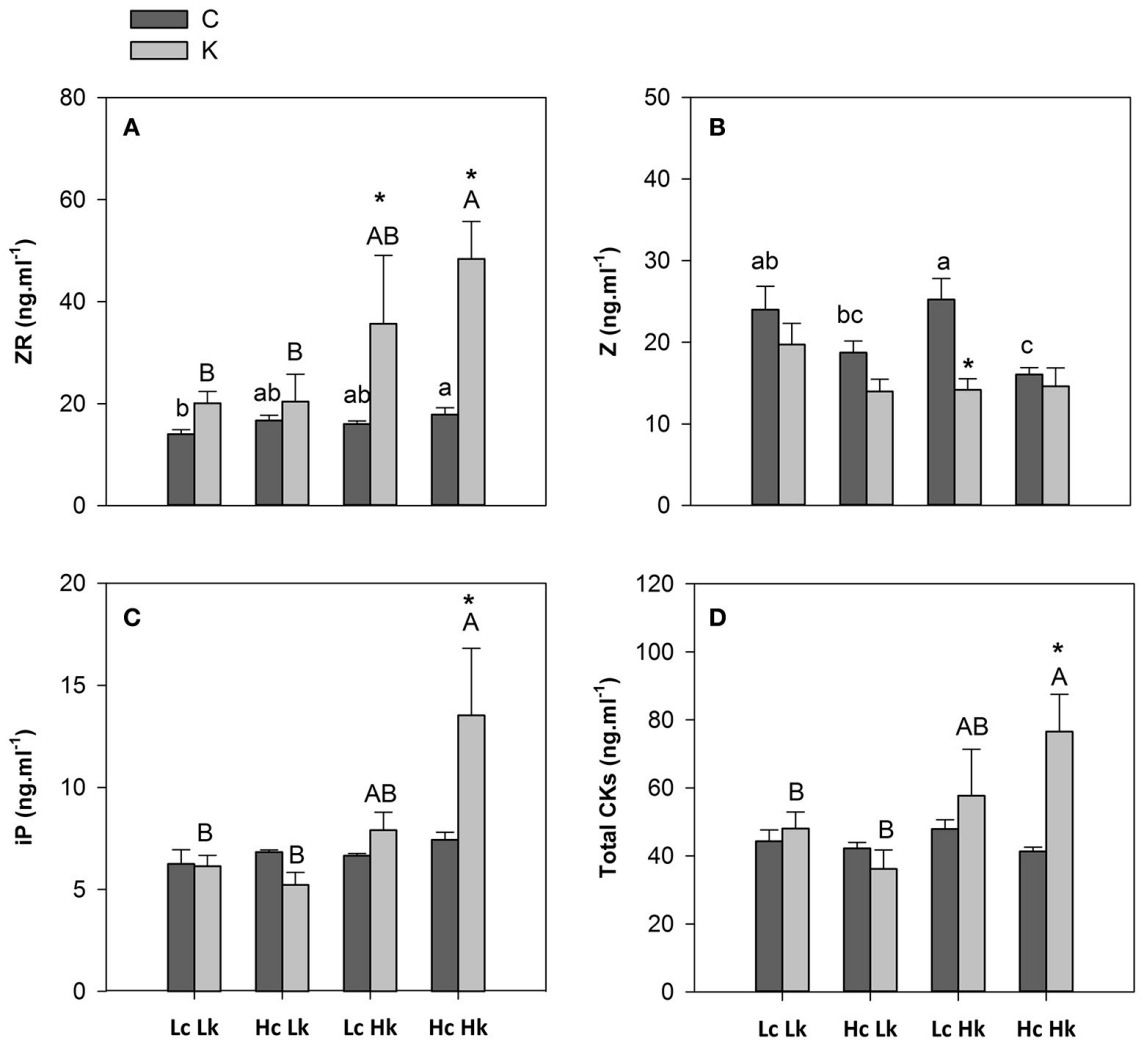
**Zeatin riboside, ZR (A)**, zeatin, Z **(B)**, isopentenyl adenine, iP **(C)** and total cytokinins, CKs **(D)** concentrations in root xylem sap of the scion (*Solanum lycopersicum* cv. Boludo F1) grafted onto a population of recombinant inbred lines (RILs) from a cross between *Solanum lycopersicum* × *Solanum pimpinellifolium* with high (*H*) or low (*L*) vigor growing under control (*c*) and low K (*k*) conditions during 48 days. Different letters indicate significant differences among graft combinations (*n* = 12, *P* < 0.05) within each treatment. ^*^ indicate significant differences between control and low-K treated plants according to the Tuckey test (*P* < 0.05).

The zeatin-type CKs were more abundant than the iP-type CKs in the root xylem sap (Figure 4). Under control K nutrition, the highest ZR concentration was observed in the high-vigor *HcHk* plants, the lowest in the low-vigor *LcLk* lines, and intermediate in the sensitive *HcLk* and tolerant *LcHk* plants (Figure 4A). Under low-K, the ZR concentration increased 2 to 2.5-fold in the tolerant *LcHk* and *HcHk* graft combinations, compared to control plants while they were non-significantly affected in the low-vigor and sensitive *Lk* plants (Figure 4A). In contrast, Z levels were higher in the low-vigor *Lc* plants (25 ng ml^−1^), while the high-vigor *HcHk* plants registered the lowest values (15 ng ml^−1^) under control K nutrition (Figure 4B). Indeed, Z concentration was negatively correlated with SFW under control conditions (Table 2). Low-K supply only significantly reduced Z levels in the tolerant *LcHk* plants, but not in the high-vigor *HcHk* ones, suggesting that a conversion of Z into ZR occurred in response to low-K in the *LcHk* plants while in the *HcHk* plants, the increase in ZR and total CKs under low-K seems to be due to *de novo* CK biosynthesis.

Non-significant differences in iP or total CKs (Z+ZR+iP) concentrations in the root xylem sap were observed among graft combinations under control conditions, while low-K supply induced an increase in both iP and total CKs only in high-vigor *HcHk* plants (Figures 4C,D).

The greatest iP, ZR, and total CKs delivery rate was found in the plants grafted onto the vigorous *HcHk* rootstocks under low-K conditions (data not shown), with a positive correlation between the flow rate of ZR, iP, and CKs to the scion and SFW under low K fertilization (Table 2).

#### 1-aminocyclopropane-1-carboxylic acid

**Figure 5:**
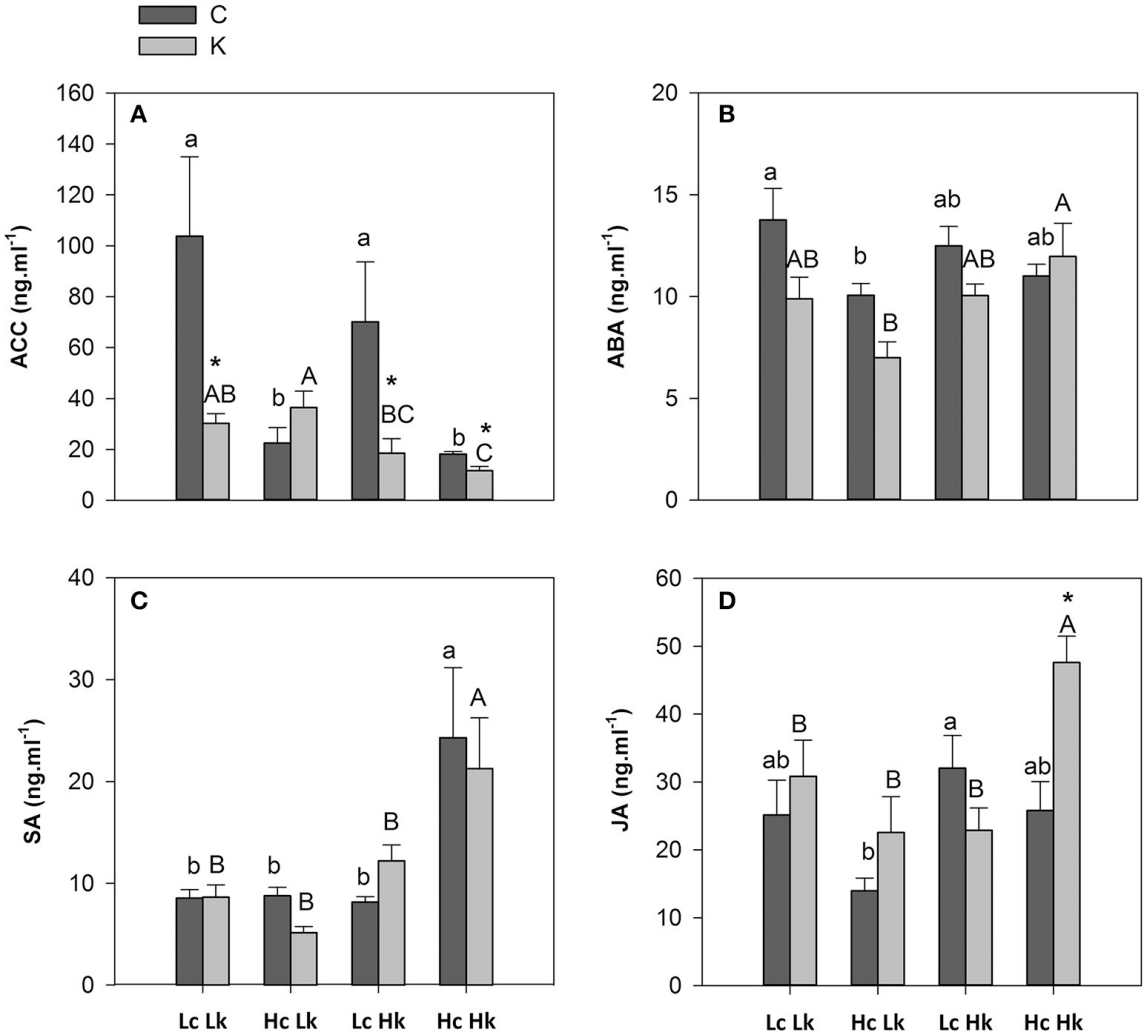
**1-Aminocyclopropane-1-carboxylic acid, ACC (A)**, abscisic acid, ABA **(B)**, salicylic acid, SA **(C)**, and jasmonic acid, JA **(D)** concentrations in the root xylem sap of the scion (*Solanum lycopersicum* cv. Boludo F1) grafted onto a population of recombinant inbred lines (RILs) from a cross between *Solanum lycopersicum* × *Solanum pimpinellifolium* with high (*H*) or low (*L*) vigor growing under control (*c*) and low K (*k*) conditions during 48 days. Different letters indicate significant differences among graft combinations (*n* = 12, *P* < 0.05) within each treatment. ^*^indicate significant differences between control and low-K treated plants according to the Tuckey test (*P* < 0.05).

Under control K nutrition, ACC concentration was significantly higher (2 to 3-fold) in the root xylem sap of the low-vigor *Lc* rootstocks than those of the high-vigor *Hc* ones (Figure 5A). Low-K supply decreased ACC concentration in the *LcLk, LcHk*, and *HcHk* plants, but the highest and lowest concentrations were registered in the low (*Lk*) and high (*Hk*) vigor rootstocks, respectively (Figure 5A). Indeed, the ACC levels were negatively correlated with the SFW under both conditions and more significantly under low-K supply (Table 2).

#### Abscisic, salicylic, and jasmonic acids

Similarly to ACC, ABA concentration in the root xylem sap under control conditions was higher in the low-vigor *Lc* rootstocks than in the high-vigor *Hc* ones, although the differences were only significant between *LcLk* and *HcLk* graft combinations (Figure 5B). Low-K supply provoked a decrease in ABA concentration in all plants except in the most vigorous *HcHk* ones, although the differences were not significant between treatments. The highest ABA levels under low-K were found in the *HcHk* graft combinations, while the lowest were registered in the sensitive *HcLk* ones (Figure 5B). Furthermore, the rootstock-mediated vigor under control conditions was negatively correlated with ABA concentrations in the root xylem sap (Table 2).

In the vigorous and tolerant *HcHk* rootstocks, SA (under control and low-K) and JA (under low-K) concentrations were twice as high as the other graft combinations (Figures 5C,D). The SA concentrations were positively correlated with SFW under low K (Table 2).

#### Gibberellins

GA_1_, GA_3_, and GA_4_ concentrations in the root xylem sap were similar in all graft combinations under control K nutrition, irrespective of their related shoot vigor (Figures S3A–D). GA_1_ and/or GA_4_ concentrations decreased under low-K supply in the different graft combinations except for the tolerant *LcHk* one (Figures S3A,C). GA_3_ was not detectable in the sensitive *HcLk* graft combination under low-K supply (Figure S3B). Although no significant correlation was found with SFW, the lowest GA concentrations under low-K (50% reduction compared to control K) were detected in those rootstocks inducing high-vigor under control conditions (*HcLk, HcHk*), while those inducing low-vigor were less (*LcHk*, 22% reduction) or not (*LcLk*) affected by the low-K supply (Figure S3D). GA_3_ seems to be a particular sensitive target under low-K supply in constitutive vigorous but susceptible rootstocks to low K supply (*HcLk*).

#### Hormonal ratios

**Figure 6:**
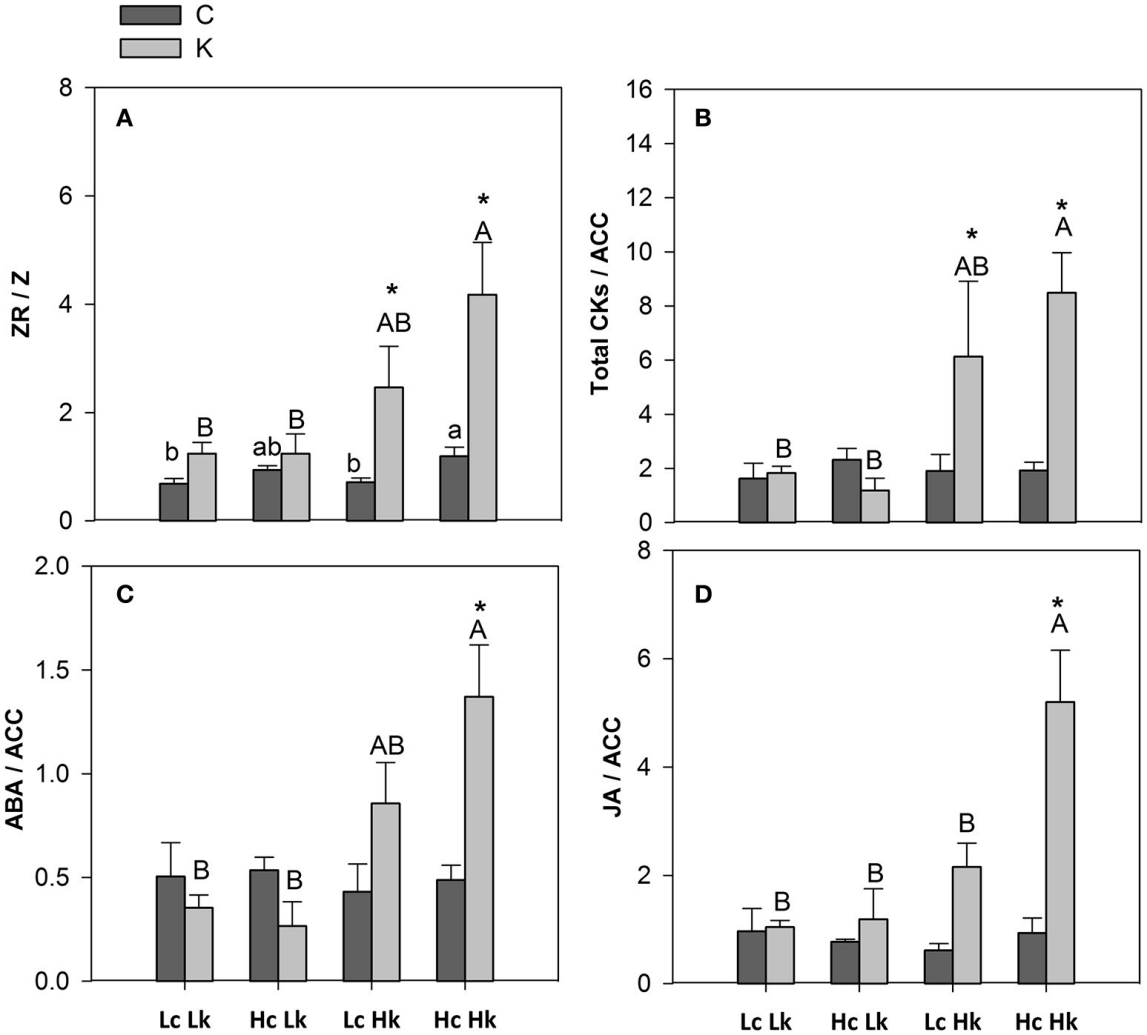
**Hormonal ZR/Z (A)**, total CKs/ACC **(B)**, ABA/ACC and JA/ACC **(D)** ratios in the root xylem sap of the scion (*Solanum lycopersicum* cv. Boludo F1) grafted onto a population of recombinant inbred lines (RILs) from a cross between *Solanum lycopersicum* × *Solanum pimpinellifolium* with high (*H*) or low (*L*) vigor growing under control (*c*) and low K (*k*) conditions during 48 days. Different letters indicate significant differences among graft combinations (*n* = 12, *P* < 0.05) within each treatment. ^*^indicate significant differences between control and low-K treated plants according to the Tuckey test (*P* < 0.05).

The ZR/Z ratio was higher in the high-vigor *Hc* graft combinations and increased under low-K in the tolerant *Hk* ones (Figure 6A). Indeed, ZR/Z was positively correlated with SFW under both control and low-K conditions (Table 2). Similarly, the CKs/ACC ratio also increased under low-K supply in the tolerant *Hk* rootstocks, registering 4–6 times higher values than the low-vigor and sensitive *Lk* ones (Figure 6B).

The ABA/ACC ratio was higher in the tolerant *Hk* graft combinations compared with the *Lk* under low-K conditions (Figure 6C). Moreover, this ratio seems to be differentially regulated by low-K in the sensitive *HcLk* (decrease) and tolerant *LcHk* (increase) graft combinations. The JA/ACC ratio significantly increased under low-K in the xylem sap of the vigorous *HcHk* plants (Figure 6D). Among the hormonal parameters analyzed, CKs/ACC, ABA/ACC, and JA/ACC ratios were the parameters most positively correlated with SFW under low-K (Table 2), suggesting that these hormonal ratios with the ethylene precursor ACC are important in determining the rootstock-mediated shoot growth under low-K conditions.

## Discussion

### Tomato rootstocks can improve shoot growth and KUE under low-K nutrition

Although grafting offers excellent opportunities to improve water and fertilizer use efficiency by exploring and exploiting the genetic diversity existing in *Solanum* spp, little is known about the physiological and genetic determinants of the rootstock capacities to improve or maintain plant growth under low nutrient supply. The use of RILs has proven to be a useful tool for these and other purposes (Albacete et al., 2009, 2015a,b; Estañ et al., 2009; Asins et al., 2010). Using 114 RILs from a *S. lycopersicum* × *S. pimpinellifolium* cross as rootstocks of a commercial tomato cultivar generate a 2.5-fold variation in the vegetative growth of the scion growing under low-K supply (1 mM; Albacete et al., 2015b). The rootstock-mediated effect on shoot growth (and subsequently in applied KUE and nutrient use efficiency) under low-K supply is at least partially due to the constitutive plant vigor observed under control conditions, as supported by (i) the positive correlation between both conditions (Figure S1) and (ii) the behavior of the low-vigor *LcLk* and high-vigor *HcHk* rootstocks (Figures 1A,B). However, the fact that the scion variety grafted onto different RILs as rootstocks can decrease (sensitive, *HcLk*) or increase (tolerant, *LcHk*) their shoot growth under low-K supply (Figure 1A) indicated that specific mechanisms control the rootstock-mediated response to low K stress in order to increase both shoot growth and KUE beyond vigor. Indeed, although it is generally assumed that rootstocks have much larger and vigorous root systems allowing the grafted plants to absorb water and nutrients more efficiently as compared to non-grafted plants, this was not the case in this study since no differences in root biomass were observed and low-K tended to decrease root biomass in the tolaerant *LcHk* rootstocks (Figure 1C). Although changes in root system architecture under low-K cannot be excluded, other more specific traits such as changes in nutrient uptake from roots and root-to-shoot transport of nutrients and hormones may help to explain the rootstock influence on the growth of the scion.

### K, Na, and Ca in the leaves and micronutrients in the root xylem sap are related to rootstock-mediated changes in shoot growth under low-K supply

Leaf K and Ca concentrations were constitutively higher in the high-vigor *HcHk* grafted plants and remained more elevated under low-K also in the tolerant *LcHk* ones, with a positive correlation with shoot biomass under low-K (Table 2) and co-varying with leaf fresh weight and *F*_v_*/F*_m_ in those suboptimal conditions (Figure 2). These results suggest that the increased Ca concentration in the leaves of those high-vigor *Hk* plants (particularly in the tolerant *LcHk* ones) could represent an important factor of the adaptive response to low-K. Indeed, Ca elevation might activate many non-selective cation channels (Amtmann and Armengaud, 2007; Demidchik and Maathuis, 2007; Wang and Wu, 2013), which could help to explain the increase in the assimilation of K and other several cations in the tolerant *Hk* rootstocks.

Since micronutrient concentrations, such as Zn, Mn, B, and Fe, were higher in the root xylem sap of high-vigor plants under low-K, specific transport mechanisms in *Hk* plants could explain these differences, rather than differences in transpiration fluxes. As foliar micronutrient concentration does not differ between graft combinations, their increased levels in root xylem sap of the *Hk* plants might stimulate growth under low-K deprivation, thus increasing the nutrient use efficiency without affecting leaf concentration (Marschner, 1995). By increasing availability in the soil or by inducing the number and/or the activity of transporters at the root membrane, micronutrient efficiency is genetically controlled and could improve crop yield under environmental stresses (Fageria et al., 2008). Importantly, QTLs controlling nutrient levels in leaves under moderate salinity were also found clustered in the RIL population from which some rootstocks were selected for this study (Asins et al., 2015). QTLs for Ca, Mn, Sr, and B levels collocated within a 10 cM region on chromosome 3, where several candidate genes have been previously identified (Asins et al., 2015). Favorable alleles of one or several of these QTL could be present in the tolerant *Hk* rootstocks, therefore enhancing loading of ions into the xylem from the roots (B, Mn, Zn, Fe) and nutrient assimilation in the leaves (K, Ca, S), which will increase shoot growth in low-K conditions of grafted plants. Curiously, Na in the leaves was the only element that was negatively correlated (Table 2) with shoot biomass under low-K, suggesting that Na/K interferences are involved in the specific rootstock-mediated responses. The underlying physiological and molecular mechanisms involved in those nutritional changes require further investigation.

### Does ACC interfere with nutrient uptake and transport?

Low-K supply in Arabidopsis roots quickly (within hours) stimulates ACC and ethylene production and root hair elongation (Shin et al., 2005; Wang and Wu, 2013). In this study, ACC concentration only increased in the root xylem sap of the most low-K sensitive *HcLk* plants, while it decreased in the other graft combinations after 7 weeks growing under low-K (Figure 5A). Interestingly, this decrease was more significant in those plants with high constitutive ACC levels (*LcLk* and *LcHk*), and the concentrations of this hormone was negatively correlated with both shoot biomass (Table 2) and *Fv/Fm* under low-K supply. The effect of the rootstock-ACC on the shoot performance can be explained in terms of nutrient uptake/assimilation, leaf senescence and growth regulation in coordination with other root-to-shoot transported hormones.

Ethylene interacts with Na/K transporters in mediating salt stress responses depending on the plant species. The lack of ethylene overproducer (ETO1) function enhanced tissue K (and reduces Na) status through inducing the expression of HAK high-affinity K transporters in Arabidopsis (Jiang et al., 2013). In rice, increasing ethylene levels increases Na accumulation in the shoot and salt sensitivity by inducing *OsHKT2; 1* gene (Li et al., 2014; Yang et al., 2015). The low vigor and low K concentration of *LcLk* grafted plants (with the highest Na concentrations) suggests that the high constitutive (or induced, as occurs in the sensitive *HcLk*) levels of ACC in the root xylem could negatively regulate K/Na homeostasis in tomato by interacting with Na/K transporters. The *HKT1* gene apparently controls Na/K levels in this RIL population when used as rootstocks under salinity, and the Na concentrations of cv Boludo tissues grafted on rootstocks homozygous for the *S. pimpinellifolium* allele were higher than on rootstocks homozygous for the with the *S. lycopersicum* allele (Asins et al., 2015). As salinity stress resembles low-K availability due to its interaction with Na (Pottosin et al., 2014), the improved KUE and higher shoot growth under low K nutrition observed in the tolerant *LcHk* lines could result from the inherent variation on *HKT* gene expression among the rootstock population. Although Na could contribute to the osmotic adjustment when K is limiting, our results suggest that rootstock-mediated increase in leaf Na concentration is not correlated with improved shoot growth under low-K (this study) or under moderate salinity (Asins et al., 2015). This negative ACC-K interaction is supported by the fact that the sensitive *HcLk* graft combinations were the only ones registering an increase in xylem ACC (even though non-significant) under low-K (Figure 5A) and suffered the highest reduction in leaf K concentration (Table 1, Figure 3A). However, although ACC was the most negative hormonal factor correlated with shoot biomass under low-K (Table 2), ACC in the root xylem sap was not correlated with K concentrations either in the root xylem sap or in the leaves (data not shown), suggesting that other mechanisms are controlling K nutrition, such as the reallocation between old and young leaves (Amtmann and Armengaud, 2007). In addition, the correlation analysis suggests that the negative effect of ACC was mostly related to micronutrient (B, Mn, and Zn) uptake in the root xylem sap (data not shown), which was closely correlated with shoot biomass under low-K (Table 2). Indeed, the presence of this kind of HKT transporters in the tolerant *Hk* lines (putatively activated or upregulated by low ACC-ethylene and high Ca), that can transport a wide range of monovalent and divalent cations (Lan et al., 2010), could help to explain the higher concentration not only of K but also Zn, Mn, Fe, and B in those plants, particularly under low-K.

If ACC is a key factor regulating shoot performance under low-K in tomato by acting on nutrient transporters and/or in other shoot growth-related processes in the shoot, it could be interesting to know how ACC levels are regulated in different rootstocks and its interaction with other hormones. According to PCA and correlation analysis, ACC was inversely correlated with CKs (mainly ZR), ABA, JA, and less significantly with SA (data not shown). Most of these hormones and their ratios with ACC were positively correlated with B, Mn, and Zn in the xylem (Data not shown) and with shoot biomass under low-K (Table 2).

### ACC and its interactions with other hormones seem to govern the rootstock-mediated response to low-K supply

Long-term low-K supply decreased ACC and increased ZR (to the detriment of Z), iP, total CKs, JA (and the CK/ACC, ABA/ACC, and JA/ACC ratios) in the xylem sap of the high vigor *Hk* rootstocks, and those traits clustered with shoot growth parameters suggesting an important role for these hormones in the rootstock-mediated shoot vigor under low-K (Figure 2).

CKs are produced mainly in roots and translocated to the shoot through the xylem sap, playing a key role in root-to-shoot communication (Dodd et al., 2004; Albacete et al., 2008, 2009; Ghanem et al., 2011; Ko et al., 2014). Commonly, nutrient deprivation decreases CK concentration (Dodd et al., 2004; Cherkozianova et al., 2005; Rahayu et al., 2005) but the role of CKs in K signaling is poorly understood (Nam et al., 2012). Wang et al. (2012) found that K deficiency (0.03 mM treatment for 32 days) decreased CKs (ZR- and iP-type) concentration in the root xylem sap and leaves of cotton plants grafted onto sensitive rootstocks. In this study, low-K supply only decreased Z concentration in the root xylem sap of the tolerant *LcHk* plants, while iP and/or ZR increased in the *Hk* graft combinations under low-K. Indeed, a positive correlation exists in both studies between ZR, iP, and CK delivery to the shoot and plant performance measured as photosynthetic rate (Wang et al., 2012) or biomass (this study, Table 2), as expected from the role of CKs in delaying senescence and promoting plant growth (Gan and Amasino, 1995; Kurakawa et al., 2007).

Since the ZR/Z ratio in the root xylem sap was positively correlated with shoot biomass under control and low-K conditions (Table 2), and ZR levels increased in the high-vigor *Hk* plants while Z only decreased in the tolerant *LcHk* rootstocks, it can be suggested that ZR is a major form of CK transport in the root xylem while Z becomes the major form in the leaves (Albacete et al., 2008, 2009; Ghanem et al., 2011). An interconversion between Z and its storage form ZR in the root xylem could be a mechanism to adapt growth to nutrient (K) availability. This idea is supported by the reduced root biomass in the tolerant *LcHk* and by the positive correlation between ZR/Z and leaf K concentration (data not shown). This idea has been previously reported under salinity: increases in bioactive CKs (Z, iP, and their ribosides) and ZR/Z in mature leaves, root xylem sap and fruit were positively correlated with leaf biomass, chlorophyll fluorescence, shoot growth, and fruit yield in the tomato plants (i) grafted onto high and low vigor rootstocks from a RIL population derived from *S. lycopersicum* × *S. cheesmanii* cross (Albacete et al., 2009), or (ii) overproducing CKs through the heat-inducible (*HSP70::IPT*, as a whole plant) or constitutive (*35S::IPT*, as a rootstock) overexpression of the isopentenyltransferase (*IPT*) gene in the roots (Ghanem et al., 2011). In both cases, the improved salt tolerance mediated by an increased root CK production was related to improved K nutrition. However, the improved shoot growth and leaf K nutrition of the *Hk* rootstocks cannot be explained by increased K concentration in the root xylem and/or delivery (considering the xylem flow rate) to the shoot. A more specific mechanism must be operating, since the other nutrients analyzed in the leaves did not increase in the *Hk* plants, with the only exception of Ca (Table 1). Indeed, Ca signaling could explain not only the increased uptake of K but also an efficient re-location from older into young leaves by affecting K-transporters (Amtmann and Armengaud, 2007). In this regard, Nam et al. (2012), suggested that decreased CK levels and increases in ZR and ZR/Z ratio is a part of an ethylene mediated adaptive response to K deficiency in Arabidopsis.

CKs (negative) and ethylene (positive) seem to be antagonistic in regulating responses to low-K in Arabidopsis (Jung et al., 2009). A reduction in CK levels as a consequence of K deficiency (or in mutants affected in CK signaling or synthesis) allows fast and effective stimulation of ethylene-induced plant adaptation to low-K conditions (Nam et al., 2012). In our study, however, the CKs/ACC ratio was positively correlated with shoot biomass, suggesting that both hormones and the balance between them are important in determining the rootstock-effect on plant vigor at low-K supply, but with an inverse role in tomato compared to Arabidopsis (Albacete et al., 2009, 2015a). Consistent with these results, Albacete et al. (2009) found that the CKs/ACC ratio were closely correlated with leaf fresh weight and *Fv/Fm* in tomato plants grown under moderate salt stress. It is also possible that the CKs/ACC response is transient until optimal K status is recovered, thus reconciling short-term (Nam et al., 2012) and long-term (this study) results. Hence, different rootstock-mediated traits should be considered regarding those hormones: effect on K uptake in the roots (local) and on shoot growth (root-shoot communication).

K deficiency also induces JA biosynthetic genes such as those encoding lipoxygenase, allene oxide synthase, and allene oxide cyclase, suggesting an important role of JA in plant signaling in response to K deficiency (Armengaud et al., 2004; Wang and Wu, 2013). JA was lowest in the root xylem of sensitive *HcLk* plants under control conditions and increased significantly only in the high-vigor *HcHk* under low-K, suggesting that induced JA response can mediate plant response to low-K. Similarly, the tomato *res* (*root growth rescue under salinity*) mutant had high constitutive root JA and improved K nutrition especially under salt stress conditions compared to the WT (Garcia-Abellan et al., 2015). The JA/ethylene interaction must be investigated further since the JA/ACC ratio increased significantly in the tolerant *Hk* graft combinations and it was positively correlated not only with shoot biomass (Table 2) but also with Zn, Mn, and B in the root xylem sap (data not shown) under low-K supply.

SA has long been known to play a role in the induction of biotic defense mechanisms in plants; however, recent studies revealed that it also participates in abiotic stress signaling (Jayakannan et al., 2015). SA concentration was not affected by low-K but it was 2 to 3-fold higher in the vigorous *HcHk* plants in both conditions. Moreover, SA was the only hormone positively correlated with leaf K concentration (data not shown). The fact that (i) interactions between JA, ABA, and SA have been reported in response to drought in tomato (Muñoz-Espinoza et al., 2015), (ii) SA might regulate anion and cation uptake through changes in the transmembrane electrical potential, H^+^-ATPase activity and Na/K homeostasis (Jayakannan et al., 2015), and (iii) the only QTL detected in this experiment was related to the SA concentration in the rootstock-xylem sap (data not shown), guarantee further research on the role of SA in K use efficiency.

GAs were also detected in the root xylem sap and numerous studies suggest that these hormones interact with mineral nutrition (Rubio et al., 2009), as for K (Wakhloo, 1970; Chen et al., 2001), P, and Fe (Guo et al., 2015). By comparing the two contrasting graft combinations responding negatively (*HcLk*) or positively (*LcHk*) to low-K, GA_1_, GA_3_, and GA_4_ levels decreased in the former while they were not affected in the later, contrarily to that observed for ACC, which suggest that GAs and ACC could be interacting in the rootstock-mediated response to low-K, as supported by the negative correlations found between those hormones in the root xylem sap (data not shown). Applying GA_3_ enhances K uptake in rice (Chen et al. (2001), which is required for stem elongation, but also enhances micronutrient uptake, such as Fe and Mn (Guo et al., 2015), two micronutrients that are particularly high in the tolerant *LcHk* plants (Table 1).

The ABA/ACC ratio seems to positively regulate the growth response under low-K (as observed in *LcHk* and *HcHk* plants). In Arabidopsis, ABA negatively regulated the ethylene-responsive factors ERF1 and ERF6 (Cheng et al., 2013; Sewelam et al., 2013). ERF6 expression inhibits leaf growth by activating the transcription of the gibberellin2-oxidase-6 gene, resulting in inactivation of gibberellins, and DELLA stabilization under osmotic stress (Dubois et al., 2015). This model could help to explain the fact that levels of GAs and ACC in the root xylem sap under control conditions were positively and negatively related to plant growth under low-K, respectively (data not shown).

**Figure 7:**
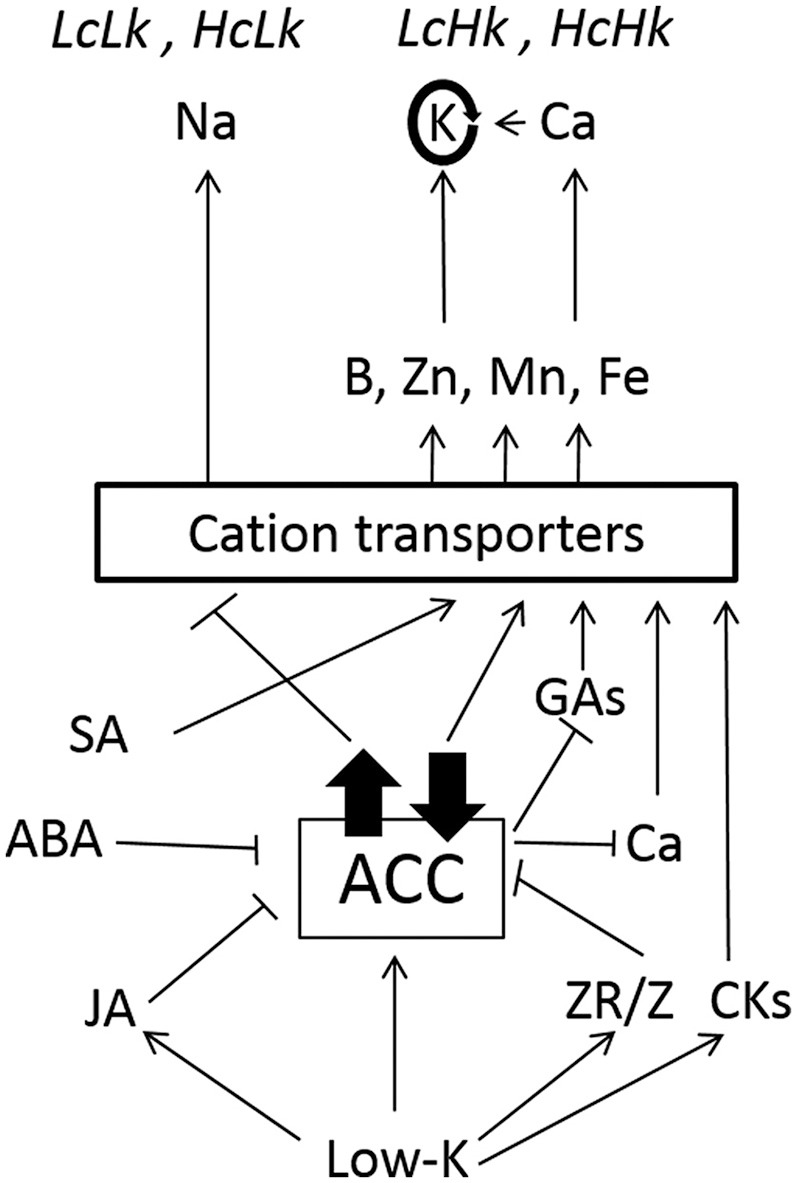
**Hormonal and nutritional interactions explaining rootstock-mediated responses to low-K supply. High constitutive or induced ACC levels would favor a higher transport of Na ***vs***. K, probably in mediating HKT transporters in ***LcLk***, ***HcLk*** plants**. Reduced Z and increased ZR/Z would contribute to decrease ACC in *LcHk* plants, thus decreasing the impact on GAs and Ca levels and improving K, B, Zn, Mn, and Fe nutrition. High constitutive SA, ABA or induced JA and CKs would contribute to low ACC activity (metabolism and/or signaling) and improved macro and micronutrition in *HcHk* plants. Arrow or bar heads indicate positive and negative regulation, respectively.

As shown in Figure 7, high ACC levels seem to favor a higher transport of Na probably in mediating cation transporters in LcLk rootstocks. The high ZR/Z ratio could contribute to reduce ACC concentrations, which may increase GA and Ca concentrations and improve micronutrients uptake in LcHk rootstocks under low-K. High constitutive SA and ABA or induced JA and CKs would help to decrease the ACC levels, thus would enhance macro and micronutrients uptake in HcHk rootstocks. Therefore, the results suggests that ACC-ethylene metabolism and signaling plays an important role by interacting with other plant hormones such as CKs, JA, SA, GAs, and ABA in the response to low-K probably by regulating ERF genes, as it seems to occur under other abiotic stresses (Müller and Munné-Bosch, 2015). These complex interactions affecting root development, nutrient uptake, root-shoot communication, and control of shoot growth and leaf senescence make it difficult to understand how the rootstock affects shoot performance but confirm grafting is a powerful tool to identify root traits for improving crops under abiotic stresses.

## Author contributions

CM-A, AA, and FP-A designed the research; CM-A, AA, and AM-P performed research; CM-A, AA, and AM-P performed data collection; CM-A and AA performed data analysis; CM-A, JP-P, and MJA performed data interpretation; JP-P and MJA performed critical revision of the article; CM-A and FP-A drafted the article; JP-P, MJA, and FP-A carried out approval of the final version.

## Funding

This research has received funding from the Spanish MINECO-FEDER (project AGL2014-59728-R) and from the European Union's Seventh Framework Programme for research, technological development and demonstration under grant agreement no 289365 (project ROOTOPOWER).

### Conflict of interest statement

The authors declare that the research was conducted in the absence of any commercial or financial relationships that could be construed as a potential conflict of interest. The reviewer AC declared a shared affiliation, though no other collaboration, with one of the authors MJA to the handling Editor, who ensured that the process nevertheless met the standards of a fair and objective review.

## Supplementary material

Table S1 **Two way ANOVA for the effects of the genotype and treatment on shoot fresh weight (SFW), K use efficiency (KUE) and K concentration in leaf (K leaf)**. The numbers in the table are F- and P-values.

Figure S1 **Shoot fresh weight (SFW) correlation between control (C) and low K condition (K) of tomato plants (***Solanum lycopersicum*** cv. Boludo F1) grafted onto a population of recombinant inbred lines (RILs) from across between ***Solanum lycopersicum*** × ***Solanum pimpinellifolium***. Lines enclosed by circles indicate the selected grafted lines within each group used in this study**. *H*, high vigor; *L*, low vigor; *c*, control conditions; *k*, low K conditions. ^**^*P* < 0.01.

Figure S2 **Sap flow of the scion (***Solanum lycopersicum*** cv. Boludo F1) grafted onto a population of recombinant inbred lines (RILs) from a cross between ***Solanum lycopersicum*** × ***Solanum pimpinellifolium*** with high (H) or low (L) vigor growing under standard (c) and low K (k) conditions during 48 days**. Different letters indicate significant differences among graft combinations (*n* = 12, *P* < 0.05) within each treatment. ^*^indicate significant differences between control and K-deprived plants accoding to the Tuckey test (*P* < 0.05).

Figure S3 **Giberellin A1 (GA1) (A)**, gibberellin A3 (GA3) **(B)** gibberellin A4 (GA4) **(C)** and total gibberellins (Total GAs) **(D)** concentrations in xylem sap of the scion (*Solanum lycopersicum* cv. Boludo F1) grafted onto a population of recombinant inbred lines (RILs) from a cross between *Solanum lycopersicum* × *Solanum pimpinellifolium* with high (H) or low (L) vigor growing under standard (c) and low K (k) conditions during 48 days. Different letters indicate significant differences among graft combinations (*n* = 12, *P* < 0.05) within each treatment. ^*^indicate significant differences between control and K-deprived plants according to the Tuckey test (*P* < 0.05).
