# Supplementary material for: Root-to-Shoot Hormonal Communication in Contrasting Rootstocks Suggests an Important Role for the Ethylene Precursor Aminocyclopropane-1-carboxylic Acid in Mediating Plant Growth under Low-Potassium Nutrition in Tomato
Source: Front Plant Sci. 2016 Nov 29;7:1782. doi: 10.3389/fpls.2016.01782 (PMC5126091; doi:10.3389/fpls.2016.01782)
Supplement: Table S1 — Two way ANOVA for the effects of the genotype and treatment on shoot fresh weight (SFW), K use efficiency (KUE) and K concentration in leaf (K leaf). The numbers in the table are F- and P-values. [file Table1.DOCX]

**Supplementary material**

**Table S1.** Two way ANOVA for the effects of the genotype and treatment on shoot fresh weight (SFW), K use efficiency (KUE) and K concentration in leaf (K leaf). The numbers in the table are *F*- and *P*-values.

|  |  | SFW (g) | | KUE (gFW. [K]^-1^) | | K leaf (mg.gDW^-1^) | |
| --- | --- | --- | --- | --- | --- | --- | --- |
|  |  | *F value* | *P value* | *F value* | *P value* | *F value* | *P value* |
| Genotype  Treatment  Genotype x treatment | | 7.295 | **0.0001** | 3.733 | **0.0001** | 4.307 | **0.0001** |
|  |  | 0.038 | 0.845 | 7.182 | 0.010 | 36.291 | **0.0001** |
|  |  | 2.345 | 0.011 | 1.359 | 0.199 | 0.783 | 0.609 |
