# Supplementary figures and images for: Root-to-Shoot Hormonal Communication in Contrasting Rootstocks Suggests an Important Role for the Ethylene Precursor Aminocyclopropane-1-carboxylic Acid in Mediating Plant Growth under Low-Potassium Nutrition in Tomato
Source: Front Plant Sci. 2016 Nov 29;7:1782. doi: 10.3389/fpls.2016.01782 (PMC5126091; doi:10.3389/fpls.2016.01782)

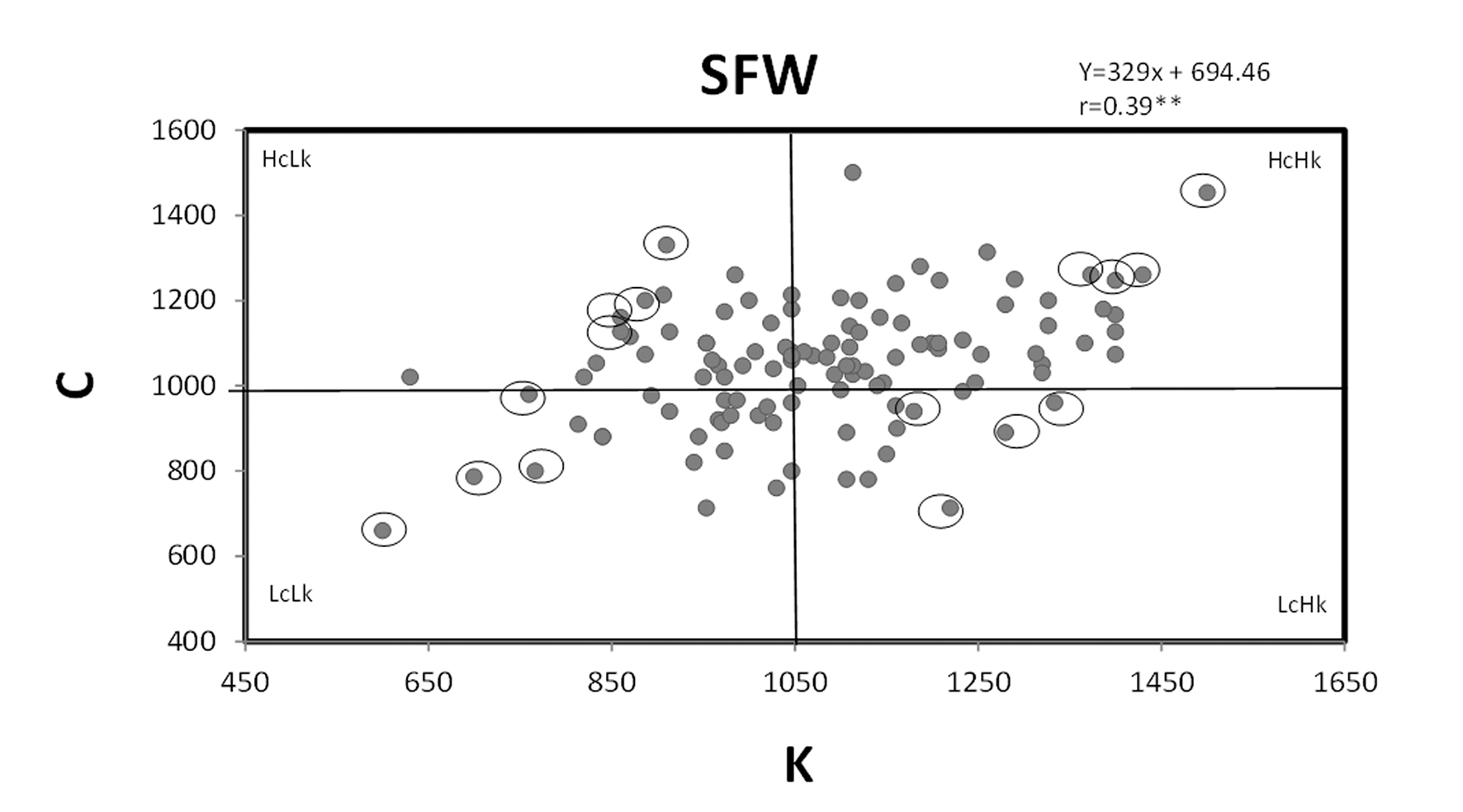

Supplement: Figure S1 — Shoot fresh weight (SFW) correlation between control (C) and low K condition (K) of tomato plants (Solanum lycopersicum cv. Boludo F1) grafted onto a population of recombinant inbred lines (RILs) from across between Solanum lycopersicum × Solanum pimpinellifolium. Lines enclosed by circles indicate the selected grafted lines within each group used in this study. H, high vigor; L, low vigor; c, control conditions; k, low K conditions. **P < 0.01. [file Image1.tif]

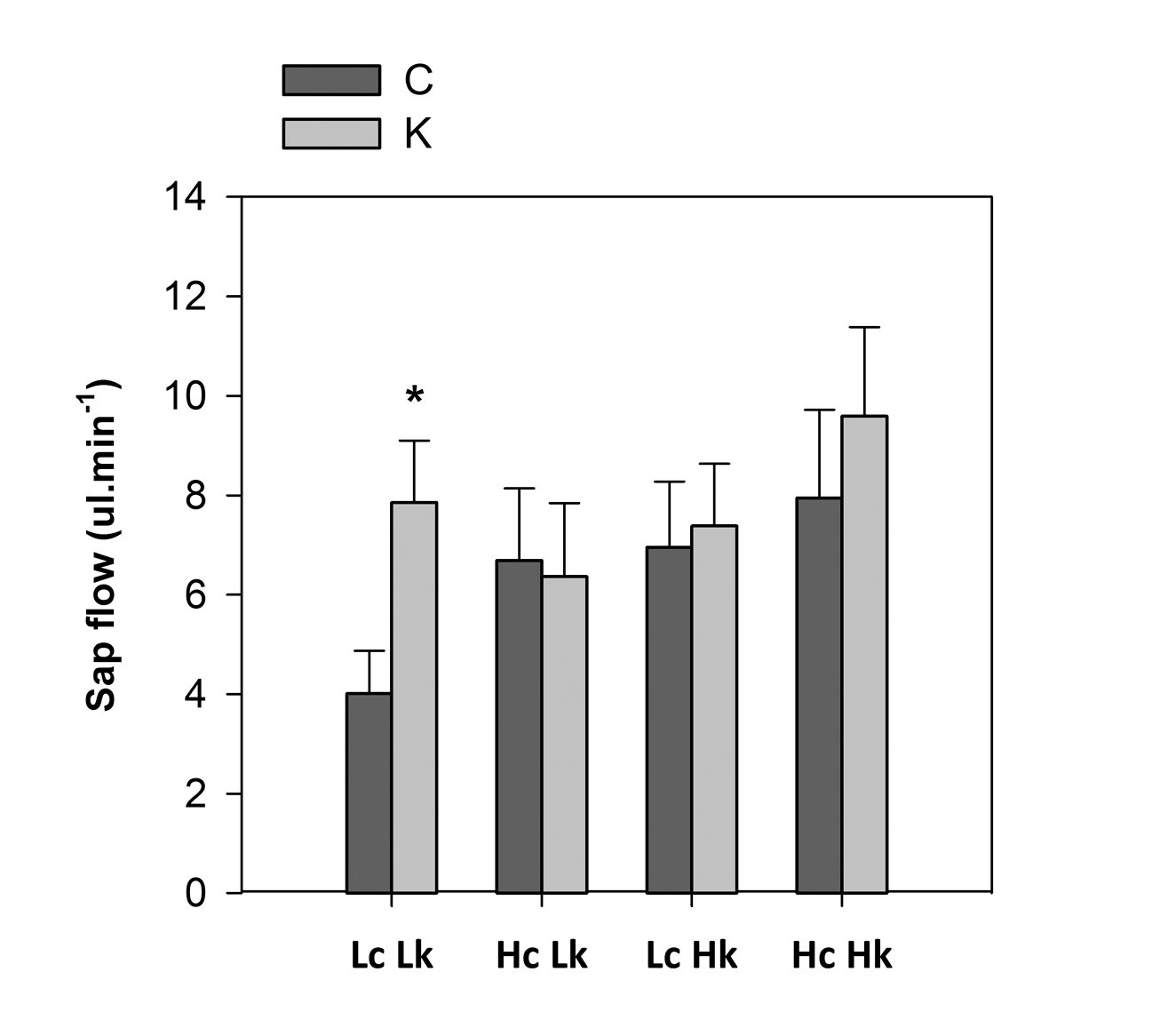

Supplement: Figure S2 — Sap flow of the scion (Solanum lycopersicum cv. Boludo F1) grafted onto a population of recombinant inbred lines (RILs) from a cross between Solanum lycopersicum × Solanum pimpinellifolium with high (H) or low (L) vigor growing under standard (c) and low K (k) conditions during 48 days. Different letters indicate significant differences among graft combinations (n = 12, P < 0.05) within each treatment. *indicate significant differences between control and K-deprived plants accoding to the Tuckey test (P < 0.05). [file Image2.tif]

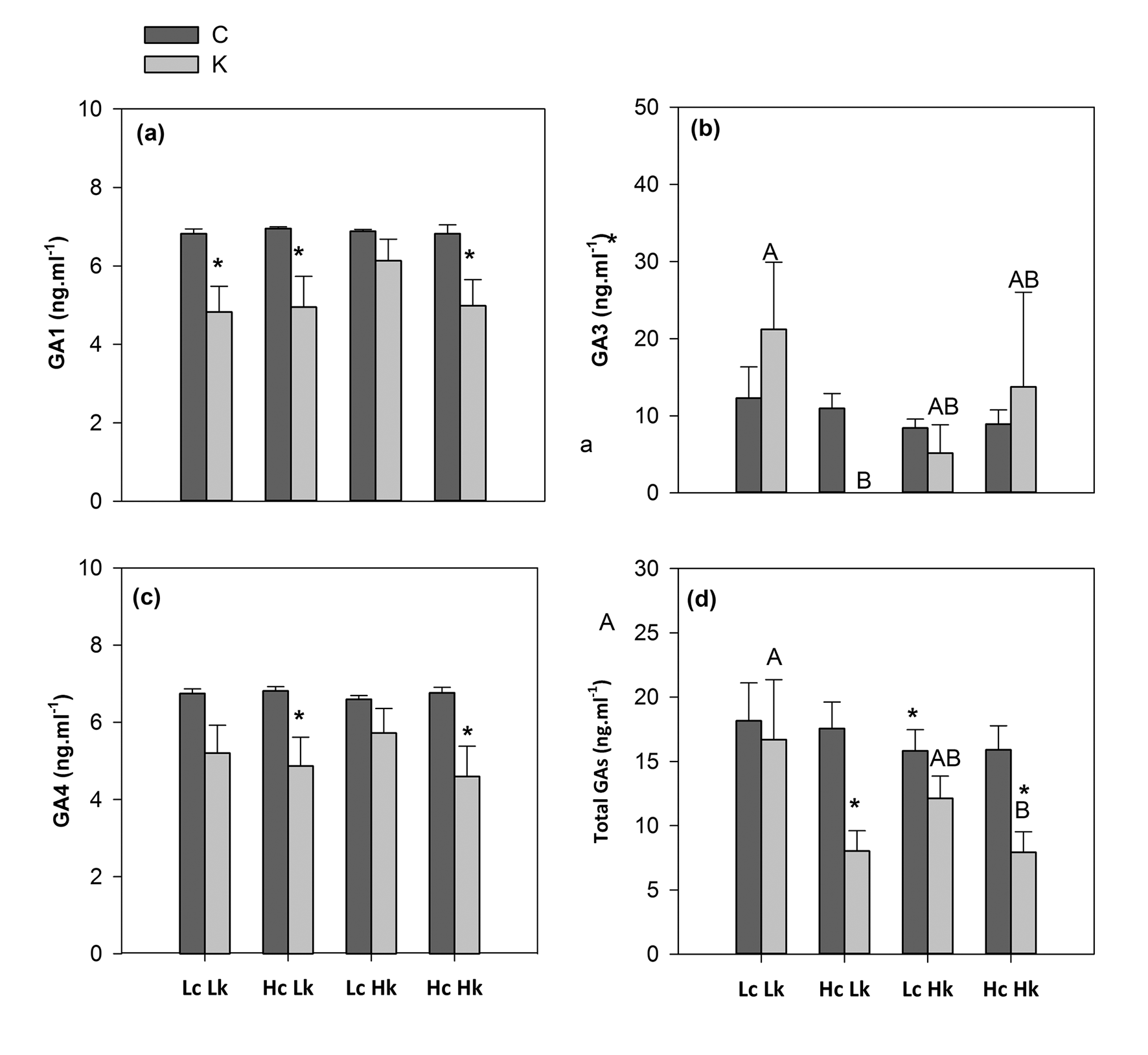

Supplement: Figure S3 — Giberellin A1 (GA1) (A), gibberellin A3 (GA3) (B) gibberellin A4 (GA4) (C) and total gibberellins (Total GAs) (D) concentrations in xylem sap of the scion (Solanum lycopersicum cv. Boludo F1) grafted onto a population of recombinant inbred lines (RILs) from a cross between Solanum lycopersicum × Solanum pimpinellifolium with high (H) or low (L) vigor growing under standard (c) and low K (k) conditions during 48 days. Different letters indicate significant differences among graft combinations (n = 12, P < 0.05) within each treatment. *indicate significant differences between control and K-deprived plants according to the Tuckey test (P < 0.05). [file Image3.tif]
